# Epidermal growth factor upregulates expression of MUC5AC via TMEM16A, in chronic rhinosinusitis with nasal polyps

**DOI:** 10.1186/s13223-020-00440-2

**Published:** 2020-05-27

**Authors:** Jian Jiao, Tao Zhang, Yu Zhang, Jingyun Li, Min Wang, Ming Wang, Ying Li, Xiangdong Wang, Luo Zhang

**Affiliations:** 1 Department of Otolaryngology, Head and Neck Surgery, Beijing TongRen Hospital, Capital Medical University, Beijing, 100730 People’s Republic of China; 2 Beijing Key Laboratory of Nasal Diseases, Beijing Institute of Otolaryngology, No. 17, HouGouHuTong, DongCheng District, Beijing, 100005 People’s Republic of China; 3 Department of Otolaryngology, Head and Neck Surgery, The Affiliated Yantai Yuhuangding Hospital of Qingdao University, Yantai, 264000 People’s Republic of China; 4 Department of Otolaryngology, Head and Neck Surgery, Tianjin Union Medical Center, Tianjin, 300121 People’s Republic of China

**Keywords:** Chronic rhinosinusitis with nasal polyps, Epidermal growth factor, Human nasal epithelial cells (HNECs), MUC5AC, Transmembrane protein 16A (TMEM16A), Phosphoinositide 3-kinase (PI3K)

## Abstract

**Background:**

Mucus hypersecretion and goblet cell upregulation are common features of chronic rhinosinusitis with nasal polyps (CRSwNP). Although epidermal growth factor (EGF) has been reported to stimulate the expression of MUC5AC, the major macro-molecular constituent of airway mucus, the precise mechanisms underlying the regulation of MUC5AC expression are still not fully understood. The aim of this study therefore was to investigate the role of EGF in regulation of mucin MUC5AC expression and define the involvement of transmembrane protein 16A (TMEM16A) in mediating the EGF-induced mucus overexpression.

**Methods:**

Human nasal epithelial cells (HNECs) derived from tissue of patients with CRSwNP and control subjects were established as air–liquid interface (ALI) cultures. Differentiated cultures were treated with different concentrations of EGF for 4–24 h, and assessed for the expression of TMEM16A and MUC5AC by real-time RT-PCR, Western blotting, ELISA and immunofluorescence. Cultures pretreated for 30 min with T16Ainh-A01 (a specific TMEM16A inhibitor) or LY294002 (a phosphoinositide 3-kinase (PI3K) inhibitor) were also assessed similarly following EGF treatment.

**Results:**

EGF treatment (10–100 ng/ml for 4–24 h) significantly increased the expression of both TMEM16A and MUC5AC mRNA and protein, as well as the percentage of TMEM16A-positive cells, MUC5AC-positive cells and cells coexpressing TMEM16A and MUC5AC in HNECs compared to control non-EGF-treated HNECs. Pretreatment of the HNECs with T16Ainh-A01 and LY294002 attenuated these EGF-induced effects.

**Conclusions:**

This study demonstrated that EGF upregulates the expression of MUC5AC in HNECs from CRSwNP patients. Furthermore, the EGF-mediated regulation of MUC5AC expression is likely to involve a PI3K-TMEM16A signalling pathway in CRSwNP.

## Background

Chronic rhinosinusitis with nasal polyps (CRSwNP) is a common chronic inflammatory disease of the upper airways, which affects 3–6% of the general population [1]. Mucus hypersecretion and goblet cell upregulation are characteristic features of CRSwNP, and partly contribute to the pathogenesis of this disease [2]. As MUC5AC is the major macro-molecular constituent of airway mucus, and because it is specifically expressed in goblet cells, regulation of MUC5AC synthesis and secretion is critical for the maintenance of goblet cell upregulation. However, the mechanisms underlying regulation of MUC5AC expression in CRSwNP are poorly understood.

Growth factors are thought to be involved in goblet cell growth, proliferation and differentiation and mucus secretion because hypersecretory diseases are associated with abnormal epithelial cell growth and differentiation, and damage to the epithelium leads to repair and remodeling processes [3]. Evidence suggests that among the growth factors, epidermal growth factor (EGF) and its receptor EGFR may be good candidates, particularly as elevated levels of EGF and EGFR have been reported in the sinus mucosa of patients with CRS and CRSwNP [4]. Furthermore, a positive correlation between EGFR expression and goblet cell hyperplasia has been shown in CRSwNP patients [2] and EGF treatment has been reported to stimulate MUC5AC expression in airway epithelial cells [5]. We have recently demonstrated that transmembrane protein 16A (TMEM16A), an important component of calcium-activated chloride channels and in the maintenance of homeostasis of the airway surface fluid in airway epithelium, is also increased together with MUC5AC in CRSwNP, possibly as a direct effect of Th2 cytokines present in sinonasal mucosa [6]. Moreover, our study demonstrated that T16Ainh-A01, a TMEM16A inhibitor, down-regulated IL-13-induced expression of both TMEM16A and MUC5AC, indicating a potentially important role of TMEM16A in mucin secretion in CRSwNP [6].

Although these studies point towards a possible involvement of EGF and TMEM16A in the regulation of mucus secretion in CRSwNP, the relationship between these mediators has not been investigated in much detail. One recent study has reported that EGF could upregulate the capacity of colonic epithelial cells to secrete Cl^−^ via upregulation of TMEM16A in the apical membrane of colonic epithelial cells [7]. In addition, this EGF-induced TMEM16A expression was mediated by sequential activation of phosphoinositide 3-kinase (PI3K) and PKCδ [7]. Thus, in view of these findings, we have hypothesized that EGF plays a role in the regulation of MUC5AC expression in CRSwNP, via a mechanism, which involves activation of PI3K and up-regulation of TMEM16A expression. The aim of the current study was therefore to investigate the effect of EGF on mucin MUC5AC expression and define the involvement of PI3K and TMEM16A in mediating the EGF-induced MUC5AC expression in primary nasal epithelial cell cultures derived from polyp tissue of CRSwNP patients.

## Methods

### Subjects

Fourteen subjects with CRSwNP (8 males, 6 females; age 18–60 years) and 4 control subjects (2 males, 2 females; age 30–60 years) without any sinonasal diseases, who presented for septoplasty for anatomic variations, were recruited for this study. A diagnosis of CRSwNP was confirmed according to the European Position Paper on Rhinosinusitis and Nasal Polyps 2012 guidelines (EPOS 2012) [8]. The diagnosis of allergic rhinitis was based on the Allergic Rhinitis and its Impact on Asthma (ARIA) guidelines-2016 revision [9]; while a diagnosis of asthma was based on the Global Initiative for Asthma 2006 guideline [10]. None of the patients had a comorbidity of allergic rhinitis, asthma or other underlying systemic disorders. Patients who had received nasal or systemic corticosteroid therapy for up to 4 weeks before surgery were excluded. Not all samples were included in every experiment because of the limited amount of some samples.

This study protocol was reviewed and approved by the ethics committee of Beijing TongRen Hospital, Capital Medical University, and written informed consent was obtained from each participant before surgery.

### Cell culture and treatments

Nasal polyp tissue from CRSwNP patients and inferior turbinate mucosa from control subjects were collected from each patient during endoscopic sinus surgery (ESS), and primary human nasal epithelial cell (HNEC) cultures were derived from the tissue as previously described [11]. Briefly, the polyp tissue was dissociated enzymatically by incubation in 0.1% protease (type XIV) (Sigma-Aldrich, St. Louis, MO, USA) in Dulbecco’s Modified Eagle Medium (DMEM) overnight at 4 °C. The isolated purified epithelial cells were collected by centrifugation at 120 g, and then seeded onto human placental collagen (Sigma-Aldrich, St. Louis, MO, USA) pre-coated, 6.5-mm-diameter polyester membrane transwells, with a pore size of 0.4 μm (COSTAR; Corning, NY, USA), at a density of 150,000 cells per well in Bronchial Epithelial Growth Medium (BEGM):DMEM (1:1) medium. Once the cells had grown to complete confluence, these were established as air–liquid interface (ALI) cultures by removing the apical medium, while adding BEGM: DMEM (1:1) medium containing 50 nM all-trans retinoic acid to the basolateral side. The ALI culture medium in the basolateral compartment was replaced every other day for a total of 14 days, by which time the cells were differentiated and the purity of epithelial cells was > 90%. Then the cells were treated basolaterally with different concentrations of EGF (10, 50, 100 ng/ml) (Sigma) for 4 h, or 24 h. In inhibition studies, cells were pretreated with a phosphatidylinositol 3-kinase (PI3K) inhibitor, LY290042 (25 μM, Cell Signaling, Danvers, MA, USA) or the TMEM16A inhibitor, T16Ainh-A01 (10 μM, Tocris, Bristol, UK) for 30 min before adding EGF and remained until termination of experiments.

### Real-time polymerase chain reaction

ALI cultures treated with EGF for 4 h were lysed in RLT buffer and total RNA was extracted using RNeasy mini kit (Qiagen, Crawley, UK), according to the manufacturer’s instructions. The purity and integrity of the total RNAs were determined by ultraviolet spectrometric measurements and agarose gel electrophoresis, respectively. Complementary DNA (cDNA) was synthesized in a 20 μl reaction volume from 1 μg total RNA using PrimeScriptTM RT Master Mix (TaKaRa Biotechnology).

Expression of TMEM16A and MUC5AC mRNA was measured with a SYBR Green method (TaKaRa Biotechnology), and relative quantitative analysis was performed using ABI7500 Real-time PCR Detection system (Applied Biosystems). The primer sequences used were as follows: (1) forward primer CCTCTGACTTCAACAGCGACAC, reverse primer TGGTCCAGGGGTCTTACTCC for housekeeping GAPDH; (2) forward primer GAAGCGGAAACAGATGCGACTC, reverse primer CTGGCTTCGTATTCAGCTCTAGG for TMEM16A; (3) forward primer AGTGTCCCCCATGCACTGA, reverse primer CAGGGGCACAAGTTCCACTG for MUC5AC.

Relative expression level of targeted genes was normalized to the mRNA level of GAPDH and quantified using the ΔΔCt method.

### Western blotting

ALI cultures treated with EGF for 24 h were harvested in cell lysis buffer, and cell lysates containing equal amounts of protein were loaded in separate lanes on 8% SDS-PAGE gels. After separation, the proteins were transferred onto nitrocellulose membrane, and non-specific binding sites were blocked by treating with 5% non-fat dry milk powder. The membrane was then incubated with primary antibodies against TMEM16A (1:1000, Abcam, Cambridge, MA, USA) overnight, followed by incubation with HRP-conjugated secondary antibody for 1 h at room temperature. Chemiluminescene was detected using the Millipore ECL kit. To confirm equal protein loading, blots were reprobed with GAPDH antibody (1:10,000). Data were analysed using the Image J software (National Institutes of Health, Bethesda, Maryland, USA).

### Muc5ac elisa

The expression of MUC5AC protein in ALI cultures treated with EGF for 24 h was analyzed by enzyme-linked immunosorbent assay (ELISA) using commercial ELISA kits (BlueGene, Biotech, Shanghai, China), according to the manufacturer’s instructions. Briefly, ALI cultures were trypsinized, centrifuged, resuspended in PBS and subjected to ultrasonication for 3 times. The total protein in cell lysates was estimated using the Bradford assay. 100 μl aliquots of the standards and samples were added to appropriate wells in the MUC5AC antibody pre-coated microtiter plate and mixed with 50 μl conjugate, before incubation at 37 °C for 1 h. At the end of incubation the each well was washed with the wash solution supplied by manufacturer, and on addition of 50 μl aliquots of substrate A and substrate B to each well, the samples were incubated at 37 °C for 15 min in the dark. At the end of incubation, 50 μl of stop solution was added to each well and absorbance was measured at 450 nm using an ELISA reader (Mithra LB 940; Berthold Technologies, Bad Wildbad, Germany).

### Immunocytochemical staining

ALI cultures treated with EGF for 24 h were fixed in a 50:50 mixture of methanol-acetone, permeabilized with 0.3% triton X-100, and blocked with 5% skimmed milk. Specimens were incubated overnight at 4 °C with TMEM16A antibody (1:200, Abcam) and MUC5AC antibody (1:200, Abcam), and then further incubated with secondary antibody rhodamine-conjugated goat anti-mouse IgG (1:500, Invitrogen) and fluorescein isothiocyanate-conjugated goat anti-rabbit IgG (1:500, Invitrogen). All specimens were then counterstained with 4, 6-diamidino-2-phenylinodole nuclear stain and examined by microscopy using an Olympus IX 81 confocal microscope (Tokyo, Japan). The number of TMEM16A-positive cells, MUC5AC-positive cells, and cells coexpressing TMEM16A and MUC5AC in 10 random fields of vision per culture was counted and expressed at a percentage of total cells.

### Statistical analysis

All data were expressed as mean ± SEM. Data analysis was performed with Prism Version 5.0 software (Graphpad Software, La Jolla, Calif). Differences between sets of differently treated cultures were analyzed using 1-way ANOVA, paired t test or 2-way ANOVA, followed by the Bonferroni post hoc test for multiple comparisons. Differences were considered significant at a p-value < 0.05.

## Results

### Effect of EGF on TMEM16A expression in HNECs

**Fig. 1:**
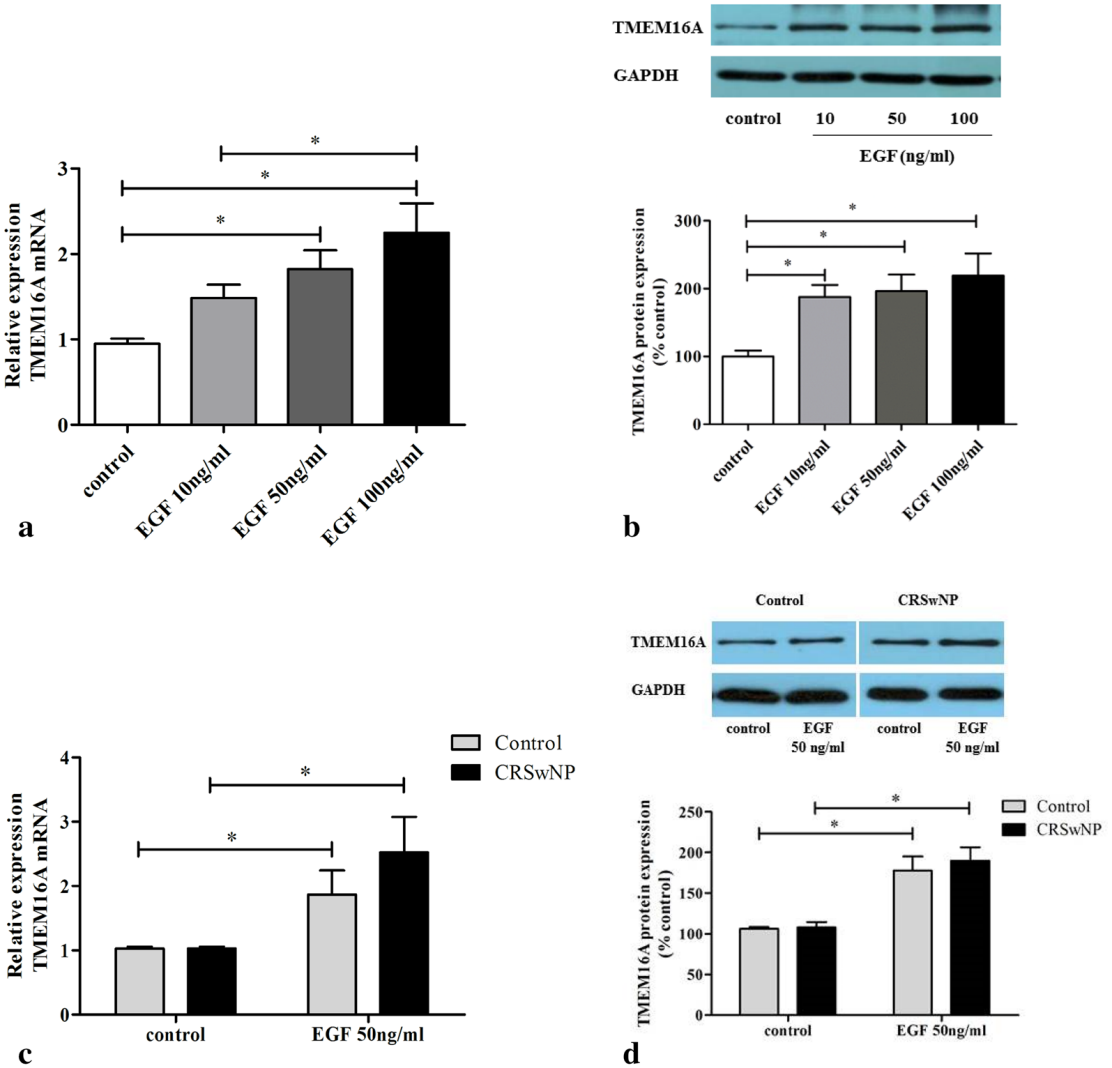
Effect of EGF on TMEM16A expression in HNECs. Cells from patients with CRSwNP (**a**, **c**) or control subjects (**c**) were treated with different concentrations of EGF for 4 h, and total RNA was analyzed by real-time RT-PCR for TMEM16A mRNA expression (n = 4); Cells from patients with CRSwNP (**b**, **d**) or control subjects (**d**) were treated with different concentrations of EGF for 24 h, and the TMEM16A protein in lysates was detected by Western blotting (n = 4). *P < 0.05, between two groups

Figure 1 shows the effect of different concentrations of EGF (10, 50, 100 ng/ml) on TMEM16A expression in HNECs. Preliminary experiments with TMEM16A and MUC5AC mRNA expression in response to EGF stimulation were determined at 4 and 24 h. The EGF-induced upregulation of TMEM16A and MUC5AC mRNA expression was stronger at 4 h than 24 h (Figures not shown). Therefore, an incubation time of 4 h was selected to assess TMEM16A and MUC5AC mRNA expression in further experiments.

As shown in Fig. 1a, incubation of HNECs derived from CRSwNP patients for 4 h with 50, 100 ng/ml EGF significantly increased the expression of TMEM16A mRNA; with 100 ng/ml EGF leading to a significant 2.2 ± 0.9-fold increase in TMEM16A mRNA, compared to control cells (p < 0.05). Western blot analysis further revealed that the expression of TMEM16A protein was also significantly increased after incubation of HNECs for 24 h with all concentrations of EGF; with the expression of TMEM16A protein significantly increased by 187.2 ± 31.3%, 196.2 ± 42.2% and 218.9 ± 56.7%, after treatment with 10, 50, and 100 ng/ml EGF, respectively, compared to control cells (Fig. 1b). Additionally, we compared the response to 50 ng/ml EGF stimulation between control culture and CRSwNP-derived culture, and found that EGF induced an increase of TMEM16A mRNA and protein in both control and CRSwNP-derived HNECs, there was no statistical difference between these cultures (Fig. 1c, d).

### Effect of EGF on MUC5AC expression in HNECs

**Fig. 2:**
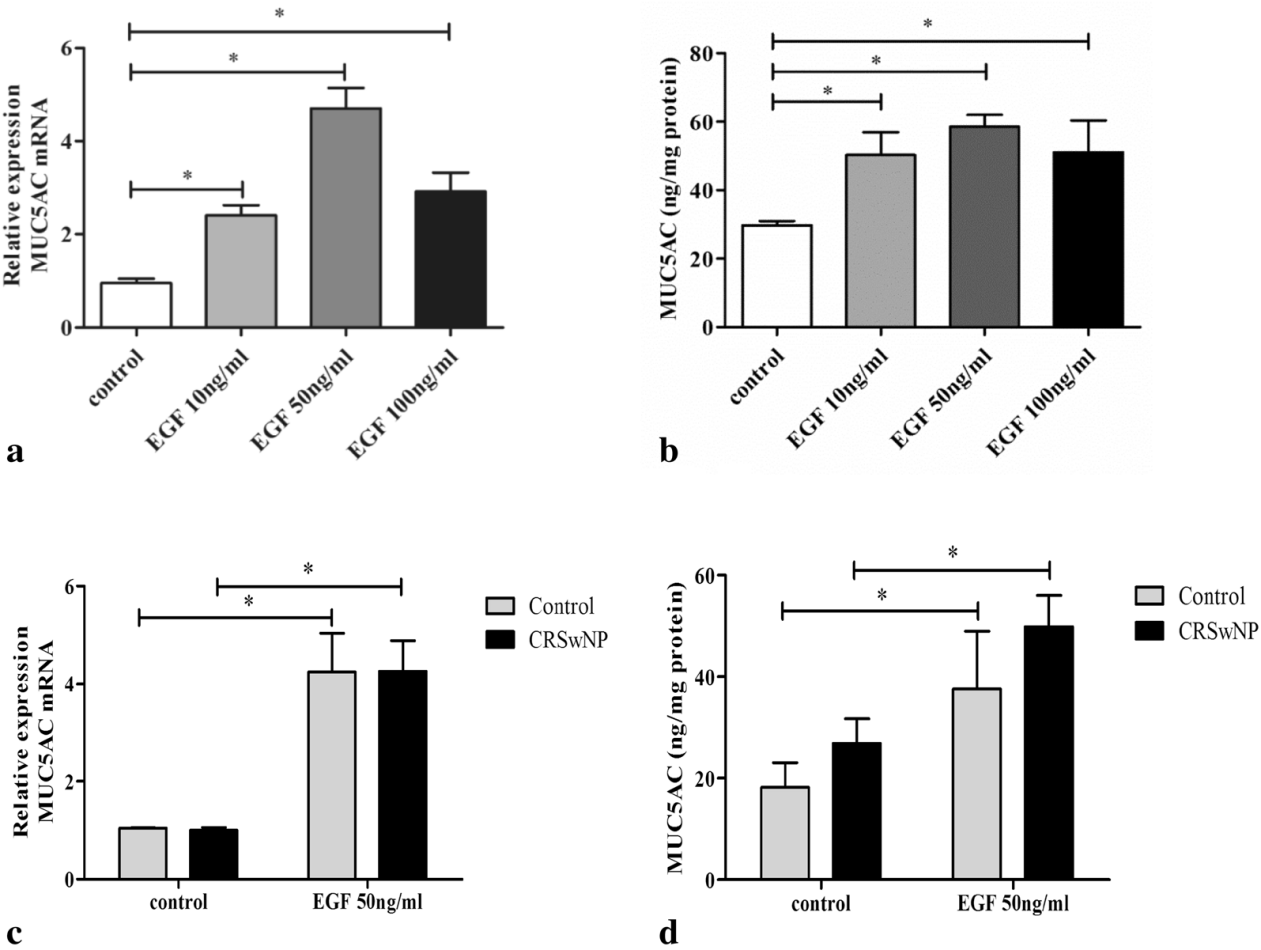
Effect of EGF on MUC5AC expression in HNECs. Cells from patients with CRSwNP (**a**, **c**) or control subjects (**c**) were treated with different concentrations of EGF for 4 h, and total RNA was analyzed by real-time RT-PCR for MUC5AC mRNA expression (n = 4); Cells from patients with CRSwNP (**b**, **d**) or control subjects (**d**) were treated with different concentrations of EGF for 24 h, and the MUC5AC protein in lysates was detected by ELISA (n = 4). *P < 0.05, between two groups

Figure 2 shows the effect of EGF treatment on the expression of MUC5AC mRNA and protein levels in HNECs. Incubation for 4 h with EGF at 10, 50 and 100 ng/ml significantly increased MUC5AC mRNA expression by 2.4 ± 0.6-fold, 4.6 ± 1.3-fold and 2.9 ± 1.2-fold, respectively, of control cells (p < 0.05 for all) in CRSwNP-derived ALI culture (Fig. 2a). Consistent with mRNA expression, the expression of MUC5AC protein was also significantly increased after incubation of the HNECs for 24 h at all concentrations of EGF (Fig. 2b). We also compared the effects of 50 ng/ml EGF on MUC5AC mRNA and protein expression between control and CRSwNP-derived culture, and found that the response to EGF stimulation was similar between the two cultures (Fig. 2c, d). Therefore, we choose CRSwNP-derived HNECs to observe the effects of PI3K or TMEM16A inhibitor on EGF-induced TMEM16A and MUC5AC expression in further experiments due to the limited number of control tissue samples.

### TMEM16A mediates EGF-induced MUC5AC expression in HNECs from CRSwNP

**Fig. 3:**
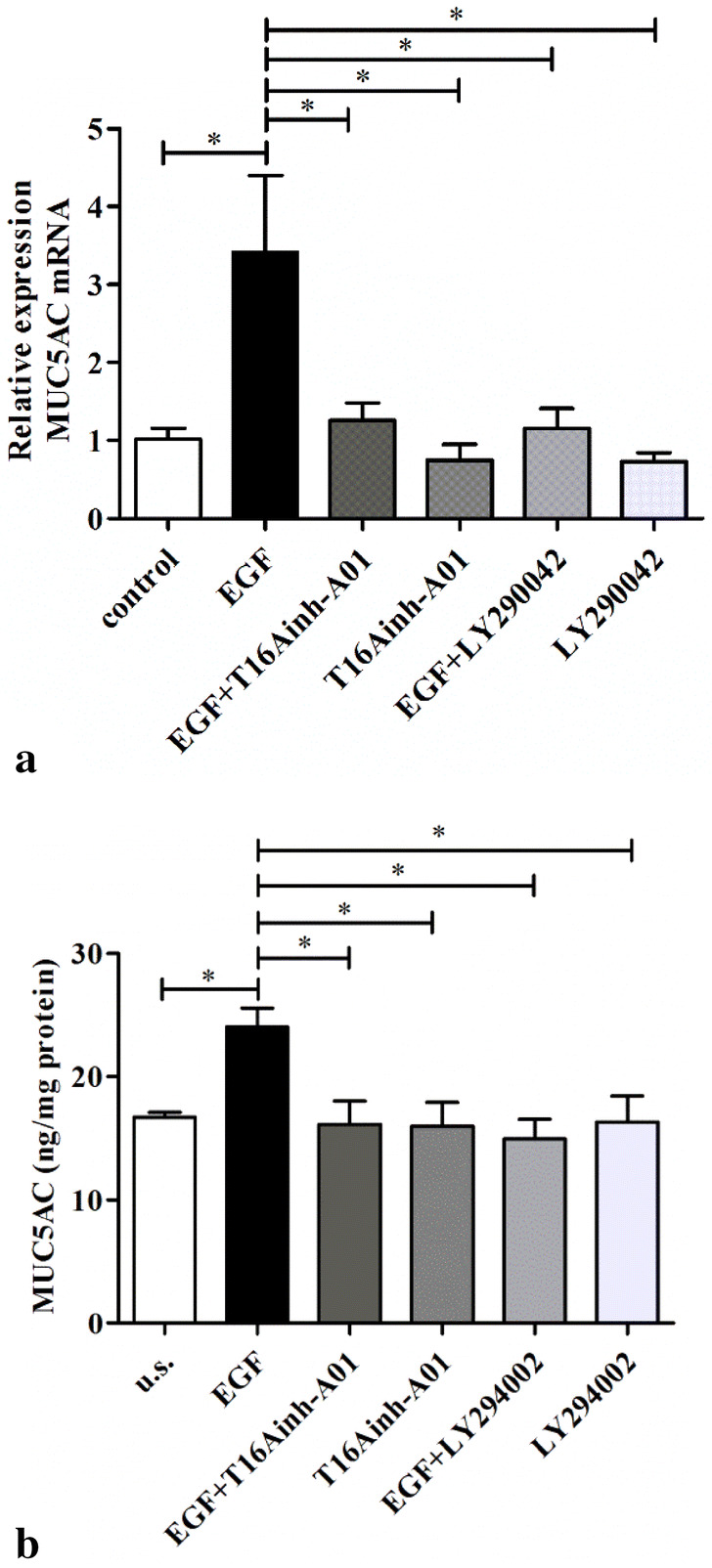
Effect of inhibitors of TMEM16A and PI3K on EGF-induced MUC5AC expression in HNECs from CRSwNP. Cells were treated with EGF (100 ng/ml) alone or with EGF following pretreatment for 30 min with T16Ainh-A01 (10 μM; the specific TMEM16A inhibitor) or with LY294002 (25 μM; the PI3K inhibitor). **a** After 4 h total RNA was analyzed by real-time RT-PCR for MUC5AC mRNA expression (n = 6); and **b** after 24 h the MUC5AC protein in lysates was detected by ELISA (n = 6). *P < 0.05, between two groups

The effect of pretreatment of HNECs with T16Ainh-A01, a specific inhibitor of TMEM16A, was investigated to determine if EGF-induced increase in MUC5AC expression was mediated by TMEM16A As shown in Fig. 3, pretreatment of the HNECs from CRSwNP patients with T16Ainh-A01 (10 μM) for 30 min before incubation with EGF 100 ng/ml significantly inhibited the EGF-induced increase in MUC5AC mRNA and protein expression; with MUC5AC mRNA and protein levels 3.4 ± 3.3-fold and 1.4 ± 0.2-fold, respectively, higher in EGF-treated HNECs and similar in T16Ainh-A01 pre-treated HNECs, compared to control cells (Fig. 3).

### Phosphatidylinositol 3-kinase (PI3K) mediates EGF-induced TMEM16A and MUC5AC expression in HNECs from CRSwNP

**Fig. 4:**
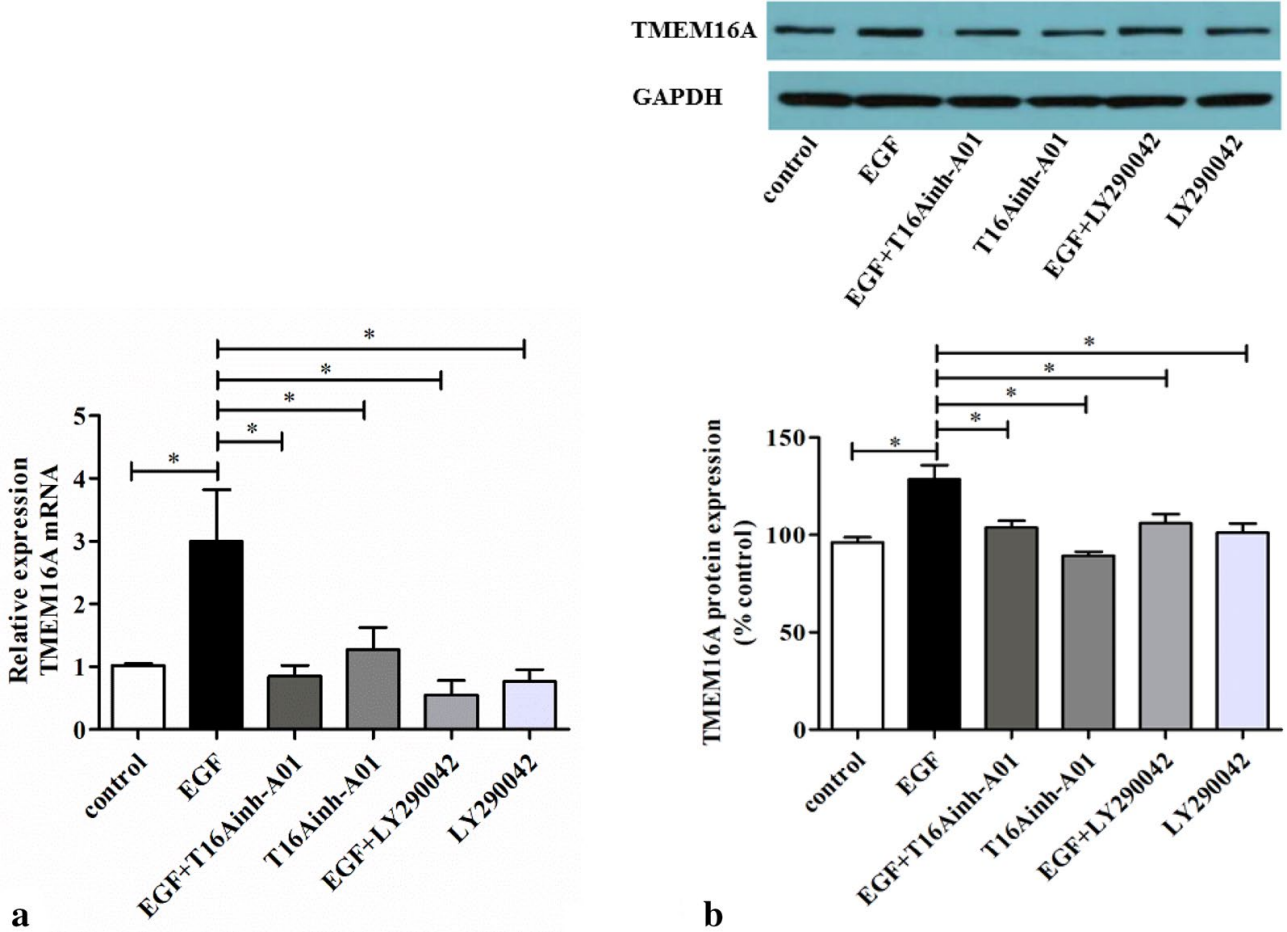
Effect of inhibitors of TMEM16A and PI3K on EGF-induced TMEM16A expression in HNECs from CRSwNP. Cells were treated with EGF (100 ng/ml) alone or with EGF following pretreatment for 30 min with T16Ainh-A01 (10 μM; the specific inhibitor of TMEM16A) or with LY294002 (25 μM; the PI3K inhibitor). **a** After 4 h total RNA was analyzed by real-time RT-PCR for TMEM16A mRNA expression (n = 6); and **b** after 24 h the TMEM16A protein in lysates was detected by Western blotting (n = 6). *P < 0.05, between two groups

To investigate the role of PI3K in mediating the increase in TMEM16A and MUC5AC expression in response to EGF in HNECs, the HNECs from CRSwNP patients were pretreated for 30 min with PI3K inhibitor, LY294002 (25 μM), before treatment with EGF. We found that when cells were treated with LY294002, EGF-induced (100 ng/ml, 4 h) increase of TMEM16A and MUC5AC mRNA expression was abolished (Figs. 3a and 4a). Similarly, PI3K inhibition significantly attenuated the increase of both TMEM16A and MUC5AC protein expression induced by treatment with 100 ng/ml EGF for 24 h (Figs. 3b and 4b). Again, LY294002 itself did not affect the basal TMEM16A or MUC5AC expression in the HNECs (Figs. 3 and 4). These results indicated that PI3K mediated the EGF-induced TMEM16A and MUC5AC expression.

### Immunocytochemical staining of TMEM16A and MUC5AC in HNECs from CRSwNP treated with EGF ± P13K and TMEM16A inhibitors

**Fig. 5:**
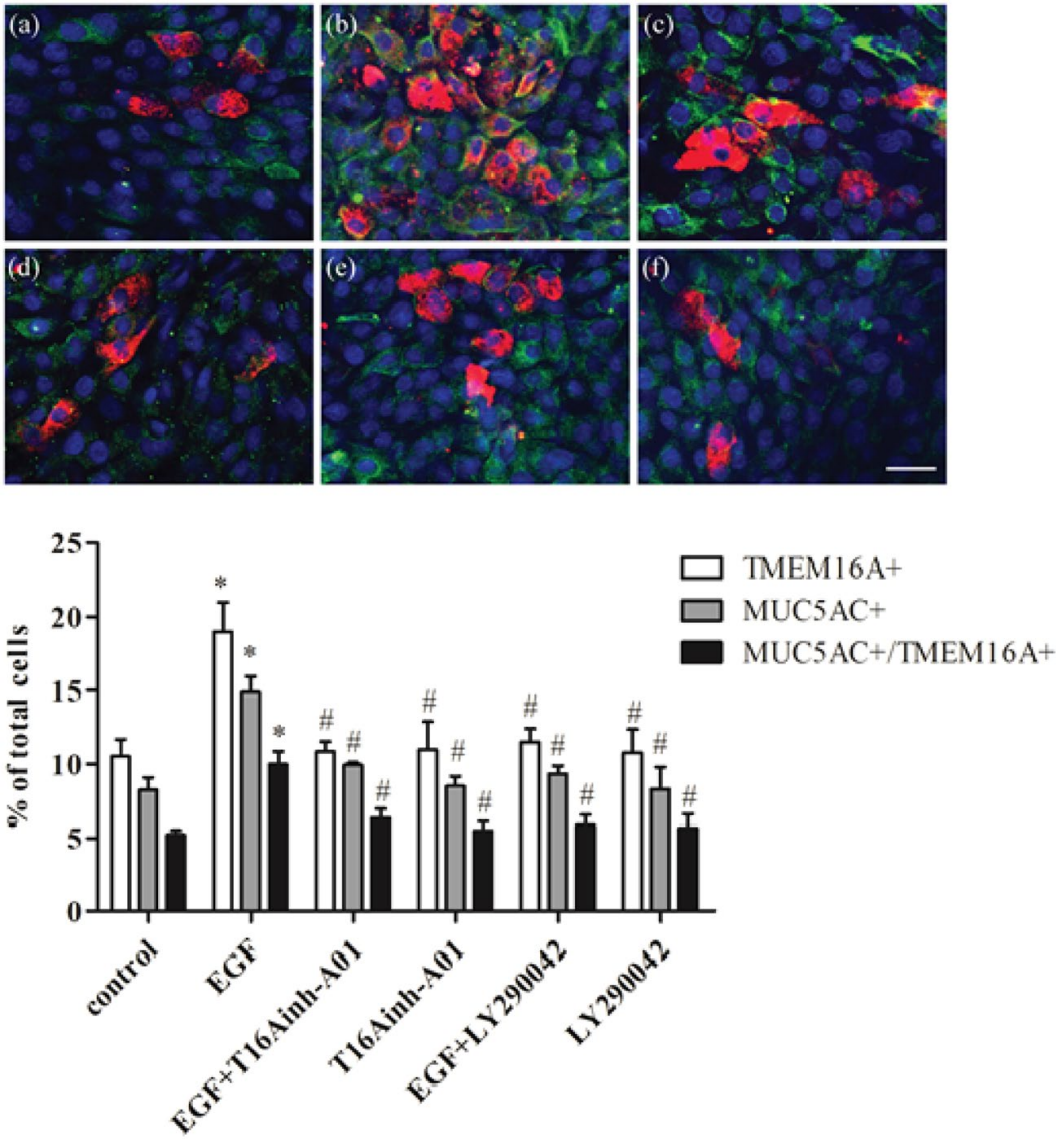
Effect of inhibitors of TMEM16A and PI3K on EGF-induced TMEM16A and MUC5AC expression in HNECs from CRSwNP. Cells were treated with EGF (100 ng/ml) alone or with EGF following pretreatment for 30 min with T16Ainh-A01 (10 μM; the specific TMEM16A inhibitor) or with LY294002 (25 μM; the PI3K inhibitor). After 24 h the cells were stained for TMEM16A and MUC5AC by immunofluorescence staining. The number of TMEM16A-positive cells, MUC5AC-positive cells, and cells coexpressing TMEM16A and MUC5AC was quantified and expressed at a percentage of total cells. Traces **a**–**f** show representative confocal images of control cells (**a**), EGF-treated cells (**b**), EGF-treated cells pretreated with T16Ainh-A01 (**c**), cells treated with T16Ainh-A01 (**d**), EGF-treated cells pretreated with LY294002 (**e**), cells treated with LY294002 (**f**). TMEM16A (green), MUC5AC (red) and nuclei (blue) are demonstrated (n = 4). Scale bar = 20 μm. *P < 0.05, vs control, #P < 0.05, vs EGF

Immunocytochemical staining for TMEM16A and MUC5AC confirmed the findings for the expression of TMEM16A and MUC5AC mRNA and protein in HNECs incubated under the different experimental conditions, described above. Thus, incubation for 24 h with EGF (100 ng/ml) of HNECs from CRSwNP showed a clear increase in the number of TMEM16A-positive cells and MUC5AC-positive cells (19.0 ± 2.8% and 14.8 ± 1.5% of total cells, respectively), compared to control cells (10.5 ± 1.6% and 8.2 ± 1.2% of total cells, respectively) (Fig. 5a, b). In addition, the proportion of coexpressing TMEM16A and MUC5AC cells incubated with EGF in HNECs (9.9 ± 1.2%) was higher than that of control cells (5.2 ± 0.4%) (Fig. 5a, b). However, the EGF-induced increase in TMEM16A-positive and MUC5AC-positive cells, as well as the percentage of cells co-expressing TMEM16A and MUC5AC, was significantly decreased in cells pre-treated with either T16Ainh-A01 or LY294002 (Fig. 5c, e), although neither inhibitor altered the percentage of TMEM16A-positive cells, MUC5AC-positive cells, and cells co-expressing TMEM16A and MUC5AC in control HNECs incubated without EGF (Fig. 5d, f).

## Discussion

**Fig. 6:**
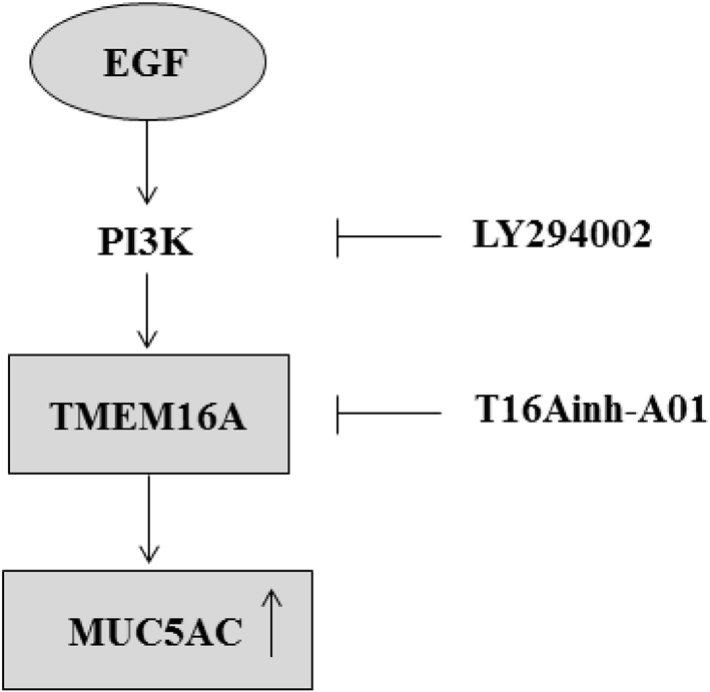
Schematic representation depicting EGF-induced MUC5AC expression via PI3K-TMEM16A pathway in HNECs from CRSwNP

Using primary HNECs derived from patients with CRSwNP and control subjects, this study has for the first time demonstrated that EGF significantly increases the expression of TMEM16A and MUC5AC at both gene and protein levels, as well as the percentage of TMEM16A-positive cells, MUC5AC-positive cells and cells coexpressing TMEM16A and MUC5AC. Furthermore, this EGF-induced upregulation of MUC5AC can be blocked by pretreatment of specific inhibitors of TMEM16A and PI3K (Fig. 6), suggesting that EGF-PI3K-TMEM16A signalling is likely to play an important role in MUC5AC expression in CRSwNP patients.

EGF is one of the multiple ligands for EGF-receptor(R), which is a 170 kDa membrane glycoprotein involved in a variety of physiological responses including cell proliferation, differentiation, motility and survival [12]. Physiologically in humans, various organs regulate their innate EGF concentrations, for example, EGF is found at high concentrations (50–500 ng/ml) in bile, urine, milk, and prostate fluid, at medium concentrations (5–50 ng/ml) in tears, follicular fluid, sperm, and seminal plasma, and at low concentrations (1–2 ng/ml) in plasma, serum, and saliva [13]. Activated EGFR has also been shown to be associated with mucin gene expression, and expressed in the airways of seasonal allergic rhinitis, COPD, cystic fibrosis, asthma, as well as CRSwNP patients [14]. Additionally, the EGF at 5–50 ng/ml was reported to be involved in the upregulation of MUC5AC expression in human airway epithelial cell lines NCI-H292 and A549, and primary rat tracheal epithelial cells [5, 15–18]. These studies strongly suggest the possible involvement of EGF signalling pathway in the regulation of mucin expression in CRSwNP. Indeed, the present study provided evidence that EGF at 10–100 ng/ml, which concentration is close to the values in vivo and reported by others [5, 15–18], directly leads to upregulation of MUC5AC expression in HNECs; thus confirming the role of EGF system in regulating goblet cell mucin expression in nasal polyps.

The present study sought to further elucidate the molecular mechanisms underlying the regulation of EGF-mediated MUC5AC expression, and demonstrated that this was likely to involve EGF-mediated upregulation of TMEM16A expression. TMEM16A is widely expressed in various types of tissues, including secretory epithelia, smooth muscle, sensory neurons and a few others, and has been reported to function as a regulator for numerous cellular functions, such as airway fluid secretion, gastrointestinal motility, exocrine glands secretion, and vascular smooth muscle contraction [19, 20]. Besides, emerging data in recent years has indicated that TMEM16A is involved in goblet cell metaplasia and mucus hypersecretion in certain airway diseases. For example, TMEM16A was found to be expressed in airway epithelium stimulated with IL-4/IL-13, and plays an important role in mucus secretion in patients with COPD and asthma [20–23]. We have previously reported that IL-13 increased the percentages of TMEM16A-positive cells, MUC5AC-positive cells, and cells coexpressing TMEM16A/MUC5AC, as well as increasing MUC5AC secretion in CRSwNP [6]. In bronchial epithelial cells, IL-4 treatment increased TMEM16A expression, mucous cell metaplasia, and the percentage of cells expressing MUC5AC [22]. In an in vitro model of well-differentiated primary normal airway epithelial cells, Huang et al. [24] showed that TMEM16A-CaCC modulates mucin secretion in response to purinergic stimulation. Similarly, recent study by Benedetto et al. [25] reported that TMEM16A was indispensable for basal mucus secretion in airways. In the current study, we have demonstrated that inhibition of TMEM16A significantly blocked EGF-induced MUC5AC upregulation and increase of MUC5AC-positive cells, suggesting that TMEM16A is also involved in mucus hypersecretion induced via the EGF/EGFR cascade.

Moreover, in the present study we also investigated the upstream pathway involved in increased TMEM16A expression and found that EGF-induced TMEM16A expression was likely to involve activation of PI3K; a lipid kinase that mediates various cellular functions, including mitogenesis, survival, motility and differentiation [26]. Indeed, some studies have reported that PI3K also plays an important role in the regulation of airway mucin production. While an early study by Atherton et al. [27] reported that PI3K was involved in IL-13-induced increase of goblet cell density in human bronchial epithelial cell cultures, a recent study by Binker et al. [28] demonstrated that LPS-stimulated MUC5AC production in NCI-H292 cells was mediated through EGFR/PI3K/Rac1 pathway. Similarly, another study by Yan et al. [26] demonstrated that the PI3K-NFAT3 pathway was involved in IL-13-induced mucus production in mouse tracheal epithelial cells. Furthermore, PI3K has been reported to be an effector protein that is activated downstream of the EGFR. In this regard Mroz et al. [7] showed that PI3K was involved in EGF-induced TMEM16A expression in colonic epithelial cells. Based on the above findings, we have hypothesized that PI3K mediates EGF-induced TMEM16A upregulation and subsequent MUC5AC expression. The present study showed that specific inhibition of PI3K also leads to blockage of EGF-induced expression of TMEM16A and MUC5AC, confirming the role of PI3K in the EGF/EGFR cascade. Thus, based on the findings of the present study, we consider that PI3K mediates EGF-induced TMEM16A upregulation and subsequent MUC5AC overexpression in the nasal passages in CRSwNP.

The molecular mechanisms underlying TMEM16A-mediated mucus hypersecretion have not been fully investigated. Lin et al. [20] have investigated TMEM16A mediated hypersecretion of mucus induced by IL-13 in human bronchial epithelial 16 (HBE16) cells, and demonstrated that the NF-κB signalling pathway may play a role and that NF-κB-mediated regulation of TMEM16A may be related to alteration of the intracellular chloride concentration. A subsequent study by these authors demonstrated that IL-13-induced over-expression of TMEM16A could induce the activation of ERK1/2 and MUC5AC generation, suggesting that activation of ERK1/2 signalling cascade may be an underlying mechanism of TMEM16A-mediated increase of mucus production [19]. Moreover, the authors demonstrated that blockade of both ERK1/2 and NF-κB pathway after transfection with TMEM16A in the HBE16 cells, did not completely block hypersecretion of mucus in these cells, suggesting that other molecular mechanisms were also likely to be involved in TMEM16A-induced mucin expression [19]. However, as distinct functions of TMEM16A have been found in different cell types, the underlying mechanisms of TMEM16A-mediated mucus production in HNECs still needs further investigation.

We have to acknowledge there are several limitations of our current study. The first one is that the number of samples used in our study is small and further confirmation with a larger population is needed. Secondly, we used the inhibitor T16Ainh-A01 to inhibit the TMEM16A, which might have non-selective effects on TMEM16A-related CaCC, further study by using TMEM16A-siRNA to knock out TMEM16A gene expression is therefore necessary. Thirdly, although we used sinonasal epithelial ALI culture system to simulate in vivo condition as closely as possible, whether the same results can be achieved in nasal tissue explant culture needs further study.

## Conclusions

In conclusion, this study has shown that treatment with the growth factor EGF upregulates the expression of MUC5AC in HNECs from CRSwNP via a pathway, which is likely to involve PI3K-TMEM16A signalling, and provides novel insights into the molecular mechanism of mucus hypersecretion in nasal polyps. Further studies will aim to confirm these findings in samples from larger number of patients and extend these findings to further elucidate the transcriptional and post-transcriptional regulation of mucin genes, which could possibly be targeted for treatment of hypersecretory diseases in the future.

## Data Availability

We would like to provide the raw data to support the information presented in this publication.

## Abbreviations

CRSwNP: Chronic rhinosinusitis with nasal polyps
EGF: Epidermal growth factor
TMEM16A: Transmembrane protein 16A
HNECs: Human nasal epithelial cells
ALI: Air-liquid interface
PI3K: Phosphoinositide 3-kinase
ESS: Endoscopic sinus surgery
DMEM: Dulbecco’s Modified Eagle Medium
BEGM: Bronchial Epithelial Growth Medium
cDNA: Complementary DNA
ELISA: Enzyme-linked immunosorbent assay
HBE16: Human bronchial epithelial 16
CRSsNP: Chronic rhinosinusitis without nasal polyp
